# A Moderated Mediation Effect of Stress-Related Growth and Meaning in Life in the Association Between Coronavirus Suffering and Satisfaction With Life: Development of the Stress-Related Growth Measure

**DOI:** 10.3389/fpsyg.2021.648236

**Published:** 2021-03-16

**Authors:** Murat Yıldırım, Gökmen Arslan

**Affiliations:** ^1^Department of Psychology, Ağri İbrahim Çeçen University, Agri, Turkey; ^2^Department of Neuroscience, Psychology and Behaviour, University of Leicester, Leicester, United Kingdom; ^3^Department of Psychological Counseling and Guidance, Mehmet Akif Ersoy University, Burdur, Turkey; ^4^International Network on Personal Meaning, Toronto, ON, Canada

**Keywords:** coronavirus suffering, meaning in life, life satisfaction, subjective well-being, stress-related growth measure

## Abstract

As previous pandemics, the coronavirus disease 2019 (COVID-19) has direct and indirect effects on mental health and well-being. The purpose of the current study was to examine whether meaning in life mediated the association between coronavirus suffering and satisfaction with life and whether stress-related growth moderated the mediating effect of meaning in life on the association between these variables. Stress-Related Growth Measure (SGM) was also conducted for the purpose of this study. The participants were 402 (66% women) young adults who completed the Suffering Measure During COVID-19, Meaningful Living Measure, Satisfaction With Life Scale, and SGM. The results indicated that the SGM has adequate psychometric properties with unidimensional structure of stress-related growth in the face of adversity. Moderated mediation analysis revealed that coronavirus suffering directly influenced satisfaction with life as well as indirectly by its effect on meaning in life. Additionally, stress-related growth was found as a moderator in the relationship between coronavirus suffering–meaning in life and coronavirus suffering–satisfaction with life. These results suggest that meaning in life mitigates the effect of coronavirus suffering on satisfaction with life, and this mediating effect is moderated by stress-related growth in young adults. While meaning in life helps explain the relationship between coronavirus suffering and satisfaction with life, the stress-related growth functions as a protective factor against the adverse effect of coronavirus experiences.

## Introduction

On January 30, 2020, the World Health Organization (WHO) has declared the novel coronavirus [coronavirus disease 2019 (COVID-19)] outbreak a public health emergency of international concern (World Health Organization, 2020). The unprecedented COVID-19 pandemic has placed an excessive degree of psychological stress on people around the globe (Arslan et al., 2020b). This pandemic has affected people's physical and mental health, and people across the globe have to cope with new psychological problems, especially with stress, anxiety, worry, uncertainty, and fear (Arslan, 2020a; Yildirim et al., 2020a,b). As the psychological impacts of the current pandemic are widespread and could affect the mental well-being of people at present and in the future, researchers have suggested urgent international research priorities, such as determining the prevalence of depression, anxiety, and other mental health problems, in various populations, for understanding mechanisms and informing interventions that affect mental well-being (Holmes et al., 2020).

According to Frankl (2000), suffering is a basic feeling that humans experience throughout the life span and an inevitable condition of life. Intrinsically, suffering has the potential to lead to a sense of displacement from others and one's own life values and goals (Svenaeus, 2014). Suffering has been conceptualized as a negative or even anguishing experience that significantly affects an individual at a psychophysical and an existential level (Bueno-Gómez, 2017; Arslan, 2021). Suffering does not always derive from illnesses or pain but also has psychological, social, and bodily dimensions. Psychosocial and physical factors such as anxiety, stress, fear, grief, painful illness, social exclusion, and forceful social inclusion can cause suffering (Bueno-Gómez, 2017). Since its emergence, COVID-19 has brought suffering, fear, anxiety, uncertainty, and death to populations across the globe. During pandemics, preventive behaviors taken by public health authorities to restrict the spread of disease can aggravate psychological, social, and spiritual suffering, which can escalate a sense of meaningless of life and even loss of faith (Schoenmaekers et al., 2020). Evidence from previous epidemics like the Middle East respiratory syndrome demonstrated a high level of suffering among populations (Hunter et al., 2016). Recent evidence suggests that people experience increased levels of suffering and pain in this health crisis, and the daily experience of suffering leads to existential distress at individuals and societal levels, which in turn has significant adverse effect on coping strategies and resilience (Rosa et al., 2020). Given the detrimental effect of suffering on the well-being and mental health of individuals, it is important to prevent individual and collective sufferings during and after the COVID-19 pandemic. Therefore, identifying how suffering is associated with well-being and its indicators plays a key role for the protection of mental well-being in times of adversities.

Subjective well-being refers to a person's cognitive and affective evaluations of his or her life in general (Diener, 1984; Kansky and Diener, 2017). These subjective evaluations are composed of emotional reactions to events and cognitive judgments of satisfaction and fulfillment (Diener, 1984, 2012). People with a high level of subjective well-being tend to experience more positive emotions, less negative emotions, and greater satisfaction with life (Moore and Diener, 2019). A low level of subjective well-being during crises and the pandemic is expected, and this affects how well a person will positively function and cope with stressful situations (Kansky and Diener, 2017; Yildirim and Belen, 2018; Yildirim and Çelik Tanriverdi, 2020). Heightening subjective well-being may facilitate a reduction in stress, anxiety, loneliness, rumination, and acute pain of adverse life events such as bereavement and unemployment or promote functional coping strategies with acute stress, thus preventing people against depression, anxiety, substance use, or other mental health conditions (Layous et al., 2014). A meta-analysis of cross-sectional, longitudinal, and experimental research suggests that subjective well-being is associated with and likely leads to positive outcomes in various domains such as work, social and intimate relationships, mental health, and physical health (Lyubomirsky et al., 2005). Furthermore, subjective well-being was found to be associated with lower stress (Yildirim and Alanazi, 2018), and anxiety and depression (Beutel et al., 2010), and higher meaning in life, resilience, and psychological health (Yildirim et al., 2021). Therefore, promoting subjective well-being is important in terms of reducing negative emotions, thoughts, and behaviors and increasing positive emotions, thoughts, and behaviors.

## Mediating Role of Meaning in Life

Although pandemic-related stress may threaten the meaning in life and undermine positive psychological health, it is possible that not all people are equally influenced. Earlier research showed that the detrimental effects of pandemic-related stress were more salient for people with low meaning in life and resilience (Yildirim et al., 2020a). However, no previous research has examined the mediating role of meaning in life in relation to coronavirus experiences, stress-related growth, and satisfaction with life in the context of the current pandemic. According to Viktor Frankl's Theory of Meaning, life has a meaning or purpose and people have a desire to find meaning in life throughout their lives (Frankl, 1969, 2000). People suffer from the “existential vacuum” when they experience negative feelings of boredom, apathy, emptiness, and depression. In this theory, by active engagement in meaningful living, people's suffering, pain, and loss can be transformed into the highest good. Meaning in life is considered one of the crucial ingredients of psychological well-being (Ryff and Keyes, 1995). A considerable body of empirical research has shown that high meaning in life is positively related to well-being as well as to a wide range of other positive outcomes (García-Alandete, 2015; Arslan et al., 2020a) and negatively related to depression, anxiety, and stress (Ishida and Okada, 2006). Research has longitudinally indicated that experience of meaning in life reduces depressive symptoms and promotes well-being (Mascaro and Rosen, 2008). Moreover, the literature has proven that meaning in life is a critical factor that mediates the impact of coronavirus-related stress and well-being and psychological health (Arslan and Yildirim, 2021; Yildirim et al., 2021) and protects mental health in the context of the pandemic (Arslan et al., 2020a). As such, promoting meaning in life can reduce pandemic-related stress and influence satisfaction with life of individuals in the face of adversity.

## Moderating Role of Stress-Related Growth

The COVID-19 pandemic emerged naturally, and its impact on mental well-being is not thoroughly known yet (August and Dapkewicz, 2020; Burke and Arslan, 2020; Yildirim and Güler, 2020). While some people experience severe mental well-being problems, others can report various positives changes such as better psychosocial adjustment and personal growth in the face of this crisis. At such times, resilient individuals can better cope with disease-related demands (Yildirim and Arslan, 2020) by experiencing stress-related growth. The term *stress-related growth* refers to positive psychological changes that people experience due to overcoming stress or adversity. Stress-related growth manifests when one copes with challenging life circumstances or events effectively. It incorporates an improved level of positive psychosocial functioning by the occurrence of positive changes and developments in one's life in comparison with a previous level. In relevant literature, similar terms such as post-traumatic growth, adversarial growth, benefit finding, and thriving as well as stress-related growth were found to be related to coping strategies and perceived stress (Schuettler and Boals, 2011), resilience (Lepore and Revenson, 2006), intrusion (Helgeson et al., 2006), psychological distress (Viegas and Henriques, 2020), and mental health and self-rated health outcomes (Drewes et al., 2020). In particular, stress-related growth was found to be associated with better psychological adjustment by increasing personal resources and positive states of mind over time (Park and Fenster, 2004). Growth following adversity was related to better psychological well-being (Durkin and Joseph, 2009). Stress-related growth was predicted by emotional processing, positive reappraisal, strength uses, and positive education (i.e., emotional management, attention and awareness, relationships, coping, and habits and goals) during a pandemic (Waters et al., 2020). This suggests that stress-related growth plays a key role in well-being and positive functioning.

## Present Study

To date, there have been few studies that report on empirical evidence on the subjective well-being of young adults during the Turkey experience of the COVID-19 pandemic or the factors (e.g., coronavirus suffering and stress-related growth) that were associated with subjective well-being at that time. Understanding the shifts in the young adults' satisfaction with life status during the COVID-19 pandemic and the factors that may be influencing changes in satisfaction with life is relevant to understanding young adults' responses to the ongoing pandemic. This is particularly important for developing the future management of pandemics. Young adulthood is an identity-forming period in which people experience a wide range of psychosocial and physical changes in emotional, behavioral, sexual, and economic areas. Young adults are considered autonomous, competent, and self-directed as well as having problem-focused coping strategies, meaningful experiences, intrinsic motivation, critical thinking ability, and decision-making skills about personal, social, and occupational roles (Bastable and Dart, 2008). Given the literature sketched above, the purpose of the current study was to examine whether meaning in life mediated the association between coronavirus suffering and satisfaction with life and whether stress-related growth moderated the mediating effect of meaning in life on the association between these variables. This study also aimed to develop and validate the Stress-Related Growth Measure (SGM) in the context of the current pandemic.

## Methods

### Participants

The study sample comprises 402 young adults (66% women) from Turkey, and they ranged in age between 20 and 48 years (*M* = 21.84, *SD* = 3.34). Regarding the coronavirus characteristics, 5% of the sample was infected. All measures and demographic items were combined, and a Web-based online survey was created. The survey was announced through social media, and people who want to participate in the study were directed to the online survey by this share. Before starting the survey, a consent form, which presented the objectives of the study and informed the students, was signed by participants. The study was also approved by Ağri İbrahim Çeçen University Institutional Review Board (ethic code: 15771).

### Measures

#### Suffering Measure During COVID-19

Suffering related to the coronavirus pandemic was assessed using the Suffering Measure During COVID-19 (SM-COVID-19; Wong, 2020) that is a 10-item self-report scale with scoring on a 5-point Likert-type scale, ranging from not at all to great deal (e.g., “Poor physical health condition”). Factor structure of the scale was affirmed using confirmatory factor analysis, which indicated an adequate data-model fit with the sample of this study: χ^2^ = 96.08, *df* = 31, *p* < 0.001, comparative fit index (CFI) = 0.96, Tucker–Lewis index (TLI) = 0.94, standardized root mean square residual (SRMR) =0.042, root mean square error of approximation (RMSEA) (95% CI) = 0.072 (0.056, 0.089). The scale had adequate to strong factor loadings, ranging between 0.44 and 0.83.

#### The Satisfaction With Life Scale

The Satisfaction With Life Scale (SWLS) was used to assess people's cognitive evaluations of their life, which is a five-item self-report scale responding with a 7-point Likert-type scale, ranging from strongly disagree to strongly agree (e.g., “I am satisfied with my life”) (Diener et al., 1985). The SWLS had a strong internal reliability estimate with a Turkish sample (Dagli and Baysal, 2016).

#### Meaningful Living Measure

Participants' sense of meaning in life was measured using the Meaningful Living Measure (MLM) that is five-item self-report scale designed to measure meaningful and purposeful living among Turkish people (Arslan, 2020b). The items of the MLM are scored using a 5-point scale, ranging from strongly disagree to strongly agree (e.g., “I find a meaning and purpose in the difficulties that I experience”). The scale had a strong internal reliability estimate (Arslan, 2020b).

#### Stress-Related Growth Measure

The SGM was developed to use in the present study. We aimed to develop and validate a brief and effective measure of stress-related growth for Turkish people. We first generated eight items based on a series of discussions within the researchers, a review of the literature, and existing self-report screeners to measure the stress-related growth of individuals. After this process, a group of three professors who work in the fields of counseling and psychology reviewed the SGM item pool. After their feedback, some revisions were made on two items to increase clarity. All pilot items of the SGM were rated using a 5-point Likert scale, ranging between 1 = *strongly disagree* and 5 = *strongly agree*. A sample item is “I become aware of the situation and focus on how I should behave.” After the item generation process, the sample of this study was randomly divided into two equal subsamples. Exploratory factor analysis was first conducted to explore the factor structure of the measure using the principal-axis factoring extraction method with Promax (oblique) rotation with the first subsample. The factor analysis results indicated that the scale items loaded on a single factor with eigenvalues >1 (3.95) that explained approximately 43% of the variance (Kaiser–Meyer–Olkin Measure of Sampling Adequacy = 0.88, Bartlett's Test of Sphericity = 1,450.13, *df* = 28, *p* < 0.001). When examining the factor leadings (≥0.50 = good factor loading), the results showed that three items had low factor loadings (Stevens, 2009; Tabachnick and Fidell, 2012), and therefore, these items were excluded and the analysis was rerun. Further results showed that the single factor accounted for 51% of the variance, with strong factor leadings (λ range = 0.64–0.83). Consistent with the eigenvalues, the parallel analysis also indicated a single-factor solution. Next, confirmatory factor analysis with Maximum Likelihood estimation using AMOS version 24.0 was performed with the second subsample of the study to affirm the emerging factor structure. The results provided excellent data-model fit statistics: χ^2^ = 7.73, *df* = 5, *p* = 0.172, CFI = 0.99, TLI = 0.98, SRMR = 0.023, RMSEA (95% CI = 0.00, 0.12) = 0.052 (Hooper et al., 2008; Mueller and Hancock, 2008). The standardized factor loadings were strong, ranging between 0.58 and 0.78, with a strong internal reliability estimate with the total sample of this study; see Table 1. Results from these analyses indicate that the SGM is a reliable and valid instrument for use to measure the growth in the face of adverse circumstances among Turkish adults.

**Table 1:** Descriptive statistics and correlations for study variables.

**Variables**	***M***	***SD***	**Skewness**	**Kurtosis**	**α**	**1**.	**2**.	**3**.	**4**.
1. Coronavirus suffering	31.86	9.69	−0.30	−0.51	0.86	–			
2. Life satisfaction	19.10	7.37	0.12	−0.69	0.84	−0.30^**^	–		
3. Meaning in life	42.44	11.79	0.34	−0.36	0.89	−0.15^**^	0.45^**^	–	
4. Stress–related growth	20.10	4.06	−1.07	1.50	0.83	−0.07	0.29^**^	0.45^**^	–

** *Correlations are significant at the 0.001 level (two-tailed)*.

### Analytic Approach

All analyses in the study were performed using SPSS version 25 and AMOS version 24.0 for Windows. As the first step of the analyses, descriptive statistics and correlation analysis were conducted to examine the assumptions of analyses and the correlations between the variables of the study. We checked the normality assumption using the kurtosis and skewness values, and their results < |2| are acceptable for a normal distribution (D'agostino et al., 1990; Field, 2009). Pearson correlation was further investigated to explore the relationships between the study variables. As the second step of the analyses, a moderated mediation analysis was employed to examine the mediating effect of meaning in life in the association between coronavirus suffering and life satisfaction and the moderating effect of stress-related growth on this mediation effect. The moderated mediation model was tested using the PROCESS macro version 3.5 (Model 8) for SPSS (Hayes, 2018) with the bootstrap approach (10,000 resamples to estimate the 95% confidence intervals) for the significance of indirect effect (Preacher and Hayes, 2008; Hayes, 2018).

## Results

### Descriptive Analyses and Correlations

Descriptive statistics, correlation analysis results, and internal reliability estimates for the variables in the present study are shown in Table 1. Reliability results indicated that all variables of the study had strong internal reliability estimates with the present sample of the study, ranging from 0.83 to 0.89 (Field, 2009). Descriptive analyses showed that skewness and kurtosis scores were between −1.07 and 1.50, demonstrating that all variables in the study had relatively normal distribution. Additional analyses indicated that coronavirus suffering had negative and significant correlations with life satisfaction and meaning in life yet had an insignificant correlation with stress-related growth. Further, life satisfaction had significant and positive correlations with meaning in life and stress-related growth, and there was a significant and positive association between meaning in life and stress-related growth, as shown in Table 1.

### Moderated Mediation Analysis

**Figure 1:**
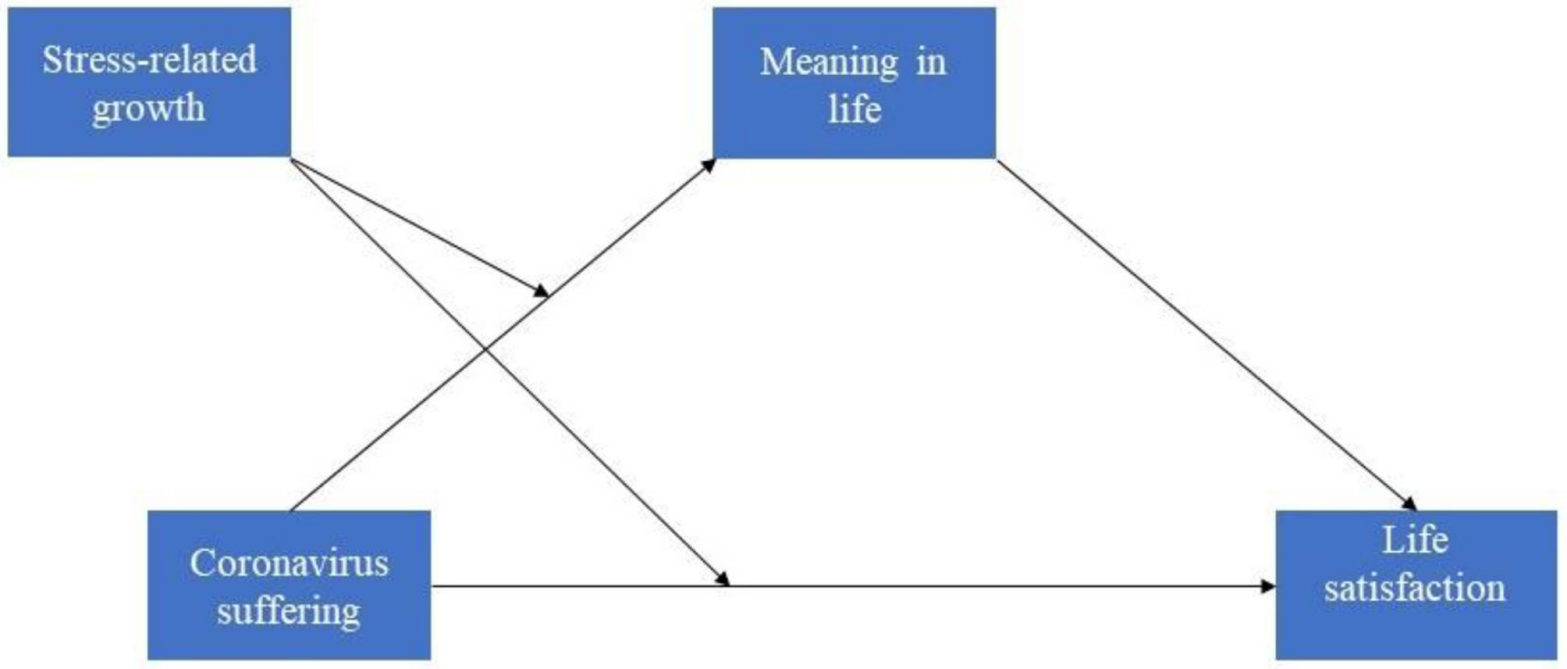
Moderated mediation model indicating the relationships between the variables.

**Figure 2:**
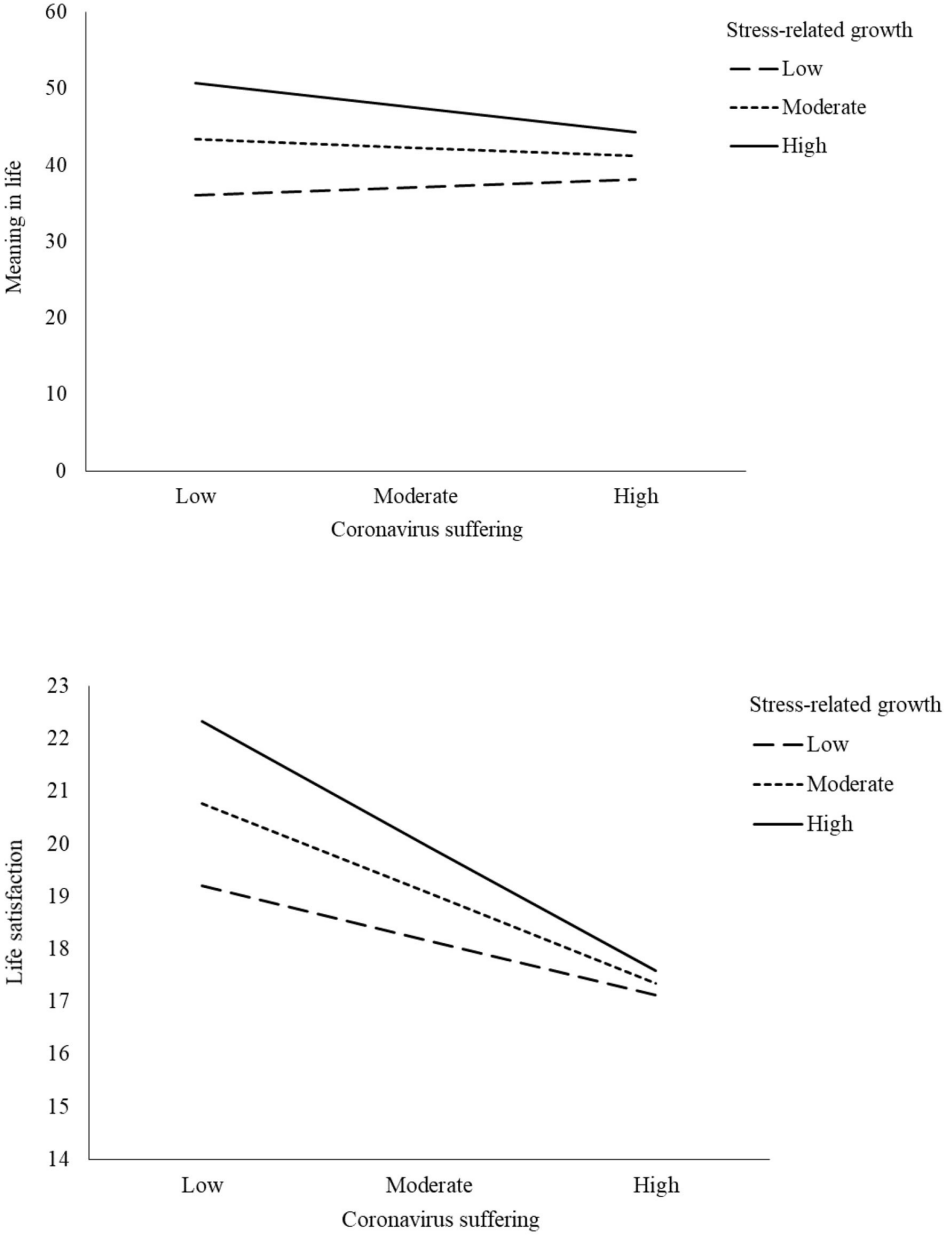
The simple slope indicating the moderation effects.

We examined whether meaning in life mediated the effect of coronavirus suffering on life satisfaction and whether stress-related growth moderated this mediating effect on life satisfaction among Turkish young adults (Figure 1). Findings from the analyses indicated that coronavirus suffering had a significant and negative predictive power on meaning in life yet did not significantly predict life satisfaction. Stress-related growth was additionally a significant and positive predictive of meaning in life, and the interaction between coronavirus suffering and stress-related growth was significant, explaining 4% of the variance in the model. The model accounted for 25% of the variance in meaning in life, as seen in Table 2. These results suggest that stress-related growth serves as a buffer in the relationship between suffering and meaning in life. The simple slope also showed that the indirect effect of coronavirus suffering on meaning in life was observed when stress-related growth was high (+1 *SD*) and moderate yet not when stress-related growth was low (−1 *SD*), as seen in Figure 2.

**Table 2:** Unstandardized coefficients for the moderated mediation model.

	**Consequent *M* (Meaning in life)**			
**Antecedent**	**Coeff**.	***SE***	***t***	***p***
*X* (Coronavirus suffering)	−0.11	0.05	−2.11	0.035
*W* (Stress-related growth)	1.28	0.12	10.19	<0.001
*X* *W	−0.05	0.01	−4.28	<0.001
Constant	42.29	0.51	82.83	<0.001
	*R*^2^ = 0.25 *F* = 44.73; *p* < 0.001			
			*Y* (Life satisfaction)	
*X* (Coronavirus suffering)	−0.18	0.03	−5.35	<0.001
*M* (Meaning in life)	0.22	0.03	6.95	<0.001
*W* (Stress-related growth)	0.23	0.09	2.53	0.011
*X* *W	0.02	0.01	−2.15	0.035
Constant	9.93	1.34	7.83	<0.001
	*R*^2^ = 0.28 *F* = 38.09; *p* < 0.001			
Conditional indirect effects of coronavirus suffering on life satisfaction				
**Stress-related growth**	**Coeff**.	**BootSE**	**BootLLCI**	**BootULCI**
*M*−1 *SD* (−3.99)	0.02	0.02	−0.02	0.06
*M* (0.00)	−0.02	0.01	−0.05	−0.01
*M* + 1 *SD* (3.99)	−0.07	0.02	−0.11	−0.04

*Level of confidence for all confidence intervals in output: 95%; Number of bootstrap samples for percentile bootstrap confidence intervals: 10,000; W (moderator variables) values in conditional tables are the mean and ±SD from the mean*.

Moderated mediation model results also showed that life satisfaction was significantly predicted by meaning in life and stress-related growth, and meaning in life significantly mitigated the effect of coronavirus suffering on life satisfaction. Further, the interaction between coronavirus suffering and stress-related growth on life satisfaction was significant, accounting for 1% of the variance in the model. The model explained 28% of the variance in life satisfaction, as shown in Table 2. These results indicate that stress-related growth project people's well-being in the face of adverse effects of coronavirus experiences. The simple slope also showed that the indirect effect of coronavirus suffering on life satisfaction was observed when stress-related growth was high (+1 *SD*), moderate, and low (−1 *SD*), as seen in Figure 2.

## Discussion

This study sought to develop our understanding of the relationships between coronavirus suffering and satisfaction with life by focusing on meaning in life as an underlying mechanism and stress-related growth as a moderator. The results showed that coronavirus suffering undermined individuals' satisfaction with life and that meaning in life played a mediating role in the associations. Stress-related growth moderated the mediating effects of meaning in life in the relationship between coronavirus stress and satisfaction with life. The following paragraphs will discuss the main contributions of the study in detail.

Firstly, our study showed that coronavirus suffering had a direct effect on individuals' satisfaction with life. Individuals who suffer from coronavirus-related stress experienced a decreased level of satisfaction with life. Although limited, the findings from previous studies showed that the experience of suffering is a prominent feeling in times of health crisis (Hunter et al., 2016). An increasing psychological suffering might induce loneliness, anxiety, stress, and fear, thereby undermining satisfaction with life. Suffering was associated with distress by decreasing the ability to cope with stress in the context of the COVID-19 pandemic (Rosa et al., 2020). Also, previous studies reported that suffering is a risk factor for diminishing well-being and preventing suffering can enhance the well-being of individuals (Quick and Henderson, 2016).

Secondly, our study demonstrated that meaning in life mediated the relationships between coronavirus suffering and satisfaction with life. A high level of coronavirus is diminished by meaning in life, which in turn leads to greater satisfaction with life. Extant studies have confirmed the positive relationships between meaning in life and satisfaction with life (García-Alandete, 2015; Arslan et al., 2020a; Yildirim et al., 2020a; Arslan and Yildirim, 2021). To the best of our knowledge, no previous study has examined the underlying relations among coronavirus suffering, meaning in life, and satisfaction with life in the context of the current pandemic. In accordance with our hypothesis, this study demonstrated that the predictive effect of coronavirus suffering on satisfaction with life was mediated by meaning in life. Therefore, this study is important in terms of advancing our knowledge about the underlying mechanism between coronavirus suffering and well-being.

Thirdly, our study showed that the direct effect of coronavirus suffering on satisfaction with life and indirect effect of coronavirus suffering on satisfaction with life *via* meaning in life were moderated by stress-related growth. This is the most noteworthy finding of this study in terms of showing the role of stress-related growth on the relationship between coronavirus suffering–meaning in life and coronavirus suffering–satisfaction with life. Stress-related growth appeared to buffer the effect of coronavirus suffering on meaning in life and satisfaction with life. Young adults with low levels of coronavirus suffering experience a greater sense of meaning in life because of experiencing stress-related growth at moderate–high levels, and they also report greater satisfaction with life because of experiencing stress-related growth at low–moderate–high levels. Furthermore, the stress-related growth moderated the mediating effect of meaning in life in the relationship between coronavirus stress and satisfaction with life. Individuals with high levels of stress-related growth are less vulnerable to coronavirus suffering and report greater meaning in life and satisfaction with life. Previous studies have demonstrated that the experience of stressors would reduce a person's resilience and the sense of meaning in life in the face of traumatic events (Yildirim et al., 2020a, 2021). Stress-related growth is related to positive states of mind, and it has benefits for individuals who experience it, including both positive changes in personal resources such as mastery and better psychological adjustment following the traumatic events (Park and Fenster, 2004). Higher growth is linked to a higher well-being (Durkin and Joseph, 2009) and other positive outcomes, including emotional processing, positive reappraisal, strength uses, and positive education (Waters et al., 2020).

Finally, the results of this study indicated that the SGM is a valid and reliable assessment tool measuring stress-related growth in the context of the COVID-19 pandemic. The scale had good psychometric properties by demonstrating adequate correlations with meaning in life and satisfaction with life with good internal consistency reliability estimate (α = 0.82). The construct validity of the SGM was established with factor analytic approach that verifies a unidimensional structure comprising five items. Although there are several stress-related growth measures in the extant literature such as Stress-Related Growth Scale (SRGS; Park et al., 1996). The original version of SRGS is excessively long and time-consuming in cases where time and resources are limited. Our measure is undoubtedly beneficial in terms of its brevity, administrability, and cost-effectiveness. The SGM can be used in subsequent research focusing on examination of stress-related growth within the context of COVID-19.

### Limitations and Implications

The present study is not without limitations that should be taken into account in further research. First, the participants mostly included women (68% women). Future research should employ the group of participants who are equally distributed in gender to increase the utility of the findings. Another limitation related to the sample was the low diversity in the sample characteristics corresponding with education level. Participants were young adults. These constraints limit the generalizability of the findings and should be taken into account in future research on a more representative sample of individuals. This suggests that both more male participation and diversity about education level should be ensured. Furthermore, participants took part in the study on a voluntary basis by completing self-report questionnaires in an uncontrolled setting. They could have been influenced by situational factors resulting in biased responses. Moreover, the current study has a cross-sectional design. Studies with longitudinal design would improve our understanding of the conditional indirect effects that relate coronavirus suffering to satisfaction with life through stress-related growth and meaning in life by identifying the potential causal and temporal relationships. Finally, future research is needed to establish further evidence of convergent validity of the SGM with similar constructs such as resilience.

Despite these limitations and suggestions about future studies, the current results reported empirical evidence that the SGM has good psychometric properties and may provide useful information about stress-related growth in the face of adversity. The development and validation of the SGM could be useful to carry out studies targeting various populations in difficult times. In addition, such a measure with good reliability and validity will be fruitful for future research and allow comparison of cross-cultural research outcomes. Most importantly, this study aimed to examine the association between coronavirus suffering and satisfaction with life by considering the mediating role of meaning in life and moderating role of stress-related growth. Based on the emerging results of the current study, stress-related growth possibly prevents people from living a meaningless life and protects their satisfaction with life in the face of adversity like the current COVID-19 pandemic, while meaning in life mitigates the effect of coronavirus suffering on satisfaction with life. For this reason, studies aimed at reducing the impact of coronavirus suffering on satisfaction with life of individuals can be organized through trainings on stress-related growth and meaning in life. For example, young adults should be informed about the possible psychological consequences of coronavirus suffering. Young adults should also be made aware that they might experience greater satisfaction with life by maintaining a greater sense of meaning in life and achieving stress-related growth even under the challenging life circumstances. To accomplish this, young adults with high coronavirus suffering should have access to psychoeducation or counseling services focusing on reduction of the adverse effect of coronavirus suffering on mental well-being.

To conclude, we found that stress-related growth moderated the mediating effect of meaning in life in the association between coronavirus stress and satisfaction with life in young adults. Stress-related growth suppressed the adverse effect of coronavirus stress on meaning in life and satisfaction with life. This study provided evidence to further develop our understanding of the conditional indirect effect by which stress-related growth influences a sense of meaning in life and satisfaction with life.

## Data Availability Statement

The raw data supporting the conclusions of this article will be made available by the authors, without undue reservation.

## Ethics Statement

The studies involving human participants were reviewed and approved by Ağri İbrahim Çeçen University Institutional Review Board. The patients/participants provided their informed consent to participate in this study.

## Author Contributions

MY and GA contributed to the design of the study. MY wrote the introduction and discussion sections. GA analyzed the data and wrote the method and results sections. Both authors contributed to manuscript revisions, read, approved the final version of the manuscript, agreed to be accountable for the content of the work, and approved the submitted version of the article.

## Conflict of Interest

The authors declare that the research was conducted in the absence of any commercial or financial relationships that could be construed as a potential conflict of interest.
