# Supplementary material for: A Moderated Mediation Effect of Stress-Related Growth and Meaning in Life in the Association Between Coronavirus Suffering and Satisfaction With Life: Development of the Stress-Related Growth Measure
Source: Front Psychol. 2021 Mar 16;12:648236. doi: 10.3389/fpsyg.2021.648236 (PMC8008138; doi:10.3389/fpsyg.2021.648236)
Supplement: Supplementary file 1 [file Table_1.DOCX]

**Stress-Related Growth Measure**

This questionnaire includes a number of statements that describe your current or past negative experiences (e.g., COVID-19, traumatic situations). Please read each item and then mark the appropriate answer in the space next to each statement. Use the rating scale from 1 (not suitable for me) to 5 (very suitable for me) to record your answers.

| 1. I am aware of the situation and focus on how I should behave. | 1 | 2 | 3 | 4 | 5 |
| --- | --- | --- | --- | --- | --- |
| 2. I empathize with others and seek how to help them. | 1 | 2 | 3 | 4 | 5 |
| 3. I live in the present moment and focus on the future. | 1 | 2 | 3 | 4 | 5 |
| 4. I look for an alternative solution to adjust to new changes. | 1 | 2 | 3 | 4 | 5 |
| 5. I pursuit for calmness, patience, and social connection. | 1 | 2 | 3 | 4 | 5 |

Note: The scale is an unidimensional structure. Sum all items to create a total score for the measure.
